# Nivolumab-related severe thrombocytopenia in a patient with relapsed lung adenocarcinoma: a case report and review of the literature

**DOI:** 10.1186/s13256-019-2245-y

**Published:** 2019-10-24

**Authors:** Takeo Hasegawa, Yuki Ozaki, Takuya Inoue, Yuzuru Watanabe, Mitsuro Fukuhara, Takumi Yamaura, Satoshi Muto, Naoyuki Okabe, Mitsunori Higuchi, Yutaka Shio, Hiroyuki Suzuki

**Affiliations:** 1 Department of Chest Surgery, Fukushima Medical University School of Medicine, 1 Hikarigaoka, Fukushima, 960-1295 Japan; 2 Department of Chest Surgery, Fukushima Medical University Aizu Medical Center, Fukushima, Japan

**Keywords:** Nivolumab, Immune checkpoint inhibitor, Non-small cell lung cancer, Immune-related thrombocytopenia

## Abstract

**Background:**

Immune checkpoint inhibitor therapy has changed the standard drug therapy for relapsed or advanced non-small cell lung cancer; its efficacy is well-recognized by pulmonary physicians, oncologists, and thoracic surgeons. Nivolumab, one of the anti-programmed cell death 1 antibodies, was the first immune checkpoint inhibitor to be approved and is used as a standard second-line regimen for patients with non-small cell lung cancer irrespective of the expression of programmed cell death ligand 1. Programmed cell death 1 antibodies have been generally confirmed to be less toxic than conventional cytotoxic chemotherapy, although unusual immune-related adverse events such as type I diabetes mellitus, adrenal failure, and myasthenia gravis may occur with a very low incidence. A case of severe grade V immune-related thrombocytopenia after two courses of nivolumab as second-line therapy for relapsed non-small cell lung cancer is reported.

**Case presentation:**

An 82-year-old Japanese woman with relapsed lung adenocarcinoma was treated with nivolumab as second-line systemic therapy at our institute. Her laboratory data indicated thrombocytopenia suspected to be an immune-related adverse event following two courses of nivolumab. Subsequently, she developed a massive pulmonary hemorrhage and left cerebral infarction despite intensive treatment including systemic steroid therapy. Although there have been a few reports of thrombocytopenia caused by nivolumab, this is the first report of grade V thrombocytopenia following administration of nivolumab for relapsed non-small cell lung cancer.

**Conclusion:**

A very difficult case of grade V immune-related thrombocytopenia after the administration of nivolumab as second-line therapy for relapsed lung adenocarcinoma was described. Immune-related thrombocytopenia is a rare adverse event, but it must be considered a possible complication because it may become critical once it has occurred.

## Background

Lung cancer is the leading cause of cancer death in the world. Platinum-based combination cytotoxic chemotherapy was the only standard treatment regimen for advanced or relapsed non-small cell lung cancer (NSCLC) for over a decade. In 2015, the programmed cell death 1 (PD-1) antibody nivolumab was confirmed to be effective and was first approved by the US Food and Drug Administration. Today, immune checkpoint inhibitors (ICIs), such as the anti-PD-1 antibodies nivolumab [1, 2] and pembrolizumab [3] or the anti-programmed cell death ligand 1 (PD-L1) antibodies atezolizumab [4] and durvalumab [5], are recognized as standard second-line therapies for advanced or relapsed NSCLC, and use of ICIs is expanding to many other malignancies. ICIs are generally recognized to be less toxic than cytotoxic chemotherapy, but they may cause unusual immune-related adverse events (ir-AEs), such as thyroiditis, type I diabetes mellitus, adrenal failure, or myasthenia gravis, while thrombocytopenia has been reported in only a few cases of advanced NSCLC [6–9]. The first case of grade V thrombocytopenia caused by nivolumab in a patient with relapsed NSCLC is presented, and the purpose of this report is to act as a warning that ICI-induced fatal thrombocytopenia could occur, although it is very rare.

## Case presentation

**Fig. 1:**
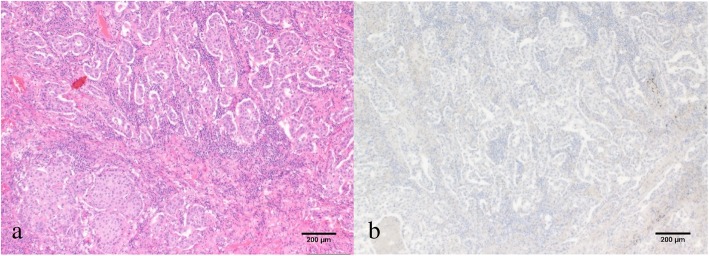
Pathological findings of the resected specimen. **a** Hematoxylin and eosin staining shows invasive adenocarcinoma of papillary predominant type. **b** Immunohistochemistry was weakly positive for programmed cell death ligand 1 (PD-L1) (SP-142 antibody)

An 82-year-old Japanese woman, who had never smoked tobacco, with a past medical history of hypertension, glaucoma, and slight renal dysfunction underwent left upper lobectomy with upper mediastinal lymph node dissection for lung cancer. She had been an office worker with telephone-related duties, and she was taking antihypertensives, proton pump inhibitors, acetaminophen, expectorants, and eye drops for glaucoma. The pathological diagnosis was T3N0M0 stage IIB adenocarcinoma with parietal pleural invasion (Fig. 1a). She had a point mutation of L858R in exon 21 of the epidermal growth factor receptor (*EGFR*) gene, and immunohistochemistry was weakly positive for PD-L1 (Fig. 1b).

**Fig. 2:**
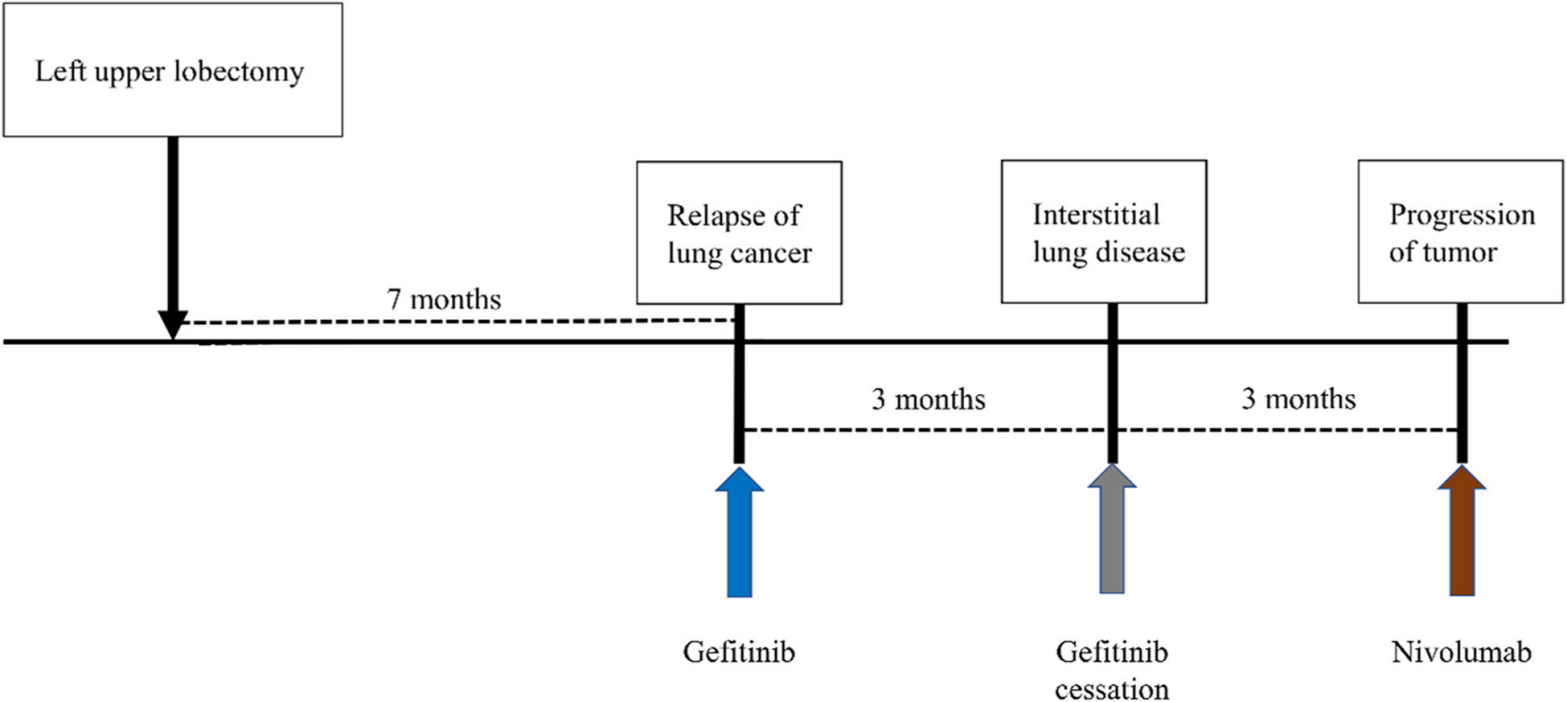
Treatment timeline after relapse of cancer. Relapse of lung cancer was detected 7 months after surgery. First-line gefitinib was discontinued due to interstitial lung disease. Three months after gefitinib was stopped, nivolumab was begun as second-line therapy

Seven months after surgery, she was diagnosed as having contralateral pulmonary metastasis and started the first-line tyrosine kinase inhibitor gefitinib. However, gefitinib was discontinued because of the development of interstitial lung disease (ILD). After cessation of gefitinib, disease progression was seen on positron emission tomography, but her Eastern Cooperative Oncology Group (ECOG) performance status was 0. Because she had left-sided back pain due to metastasis to the left pleura with chest wall invasion around the third thoracic spine, palliative irradiation to her chest wall was performed. After 30 Gy of palliative irradiation, she was given nivolumab 3 mg/kg every 2 weeks as a second-line therapy (Fig. 2). During the first two cycles of nivolumab treatment, no adverse events (AEs) were observed, and her platelet count was almost 180 × 10^3^/μL before and after administration of nivolumab. However, laboratory data before the third cycle of nivolumab showed that her platelets were extremely low (2000/μL), although her hemoglobin and white blood cell count (10.6 g/dL and 4500/μL, respectively) remained unchanged compared to the previous examination. Based on this result, she was admitted to our hospital urgently that same day.

**Fig. 3:**
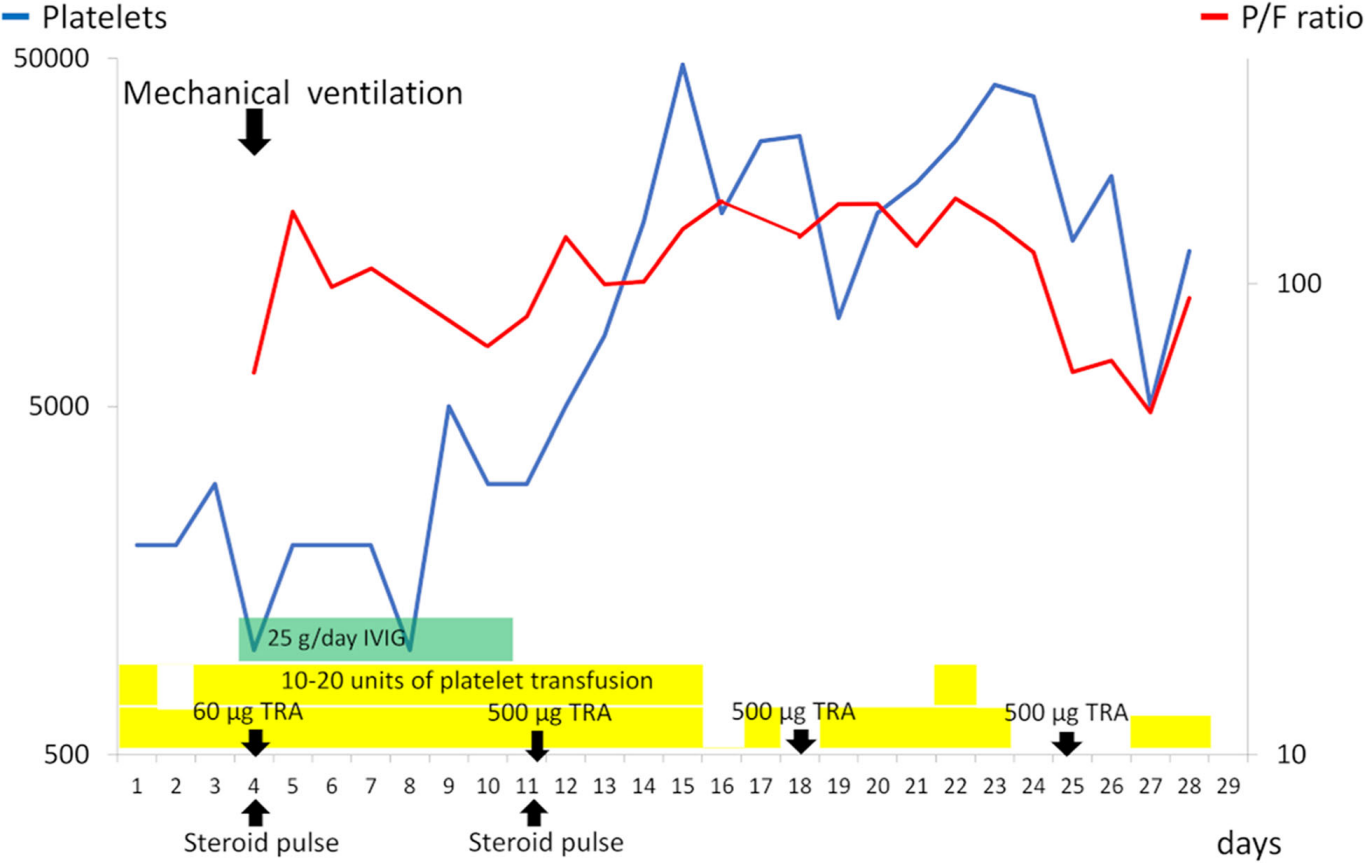
Clinical course of the present case. The platelet count recovers temporarily with intensive treatment, such as platelet transfusions, intravenous immunoglobulin, steroid pulse therapy, and romiplostim, but the patient’s general condition does not improve. *IVIG* intravenous immunoglobulin, *P/F ratio* partial pressure of oxygen in arterial blood/fraction of inspired oxygen ratio, *TRA* thrombopoietin receptor agonist

**Fig. 4:**
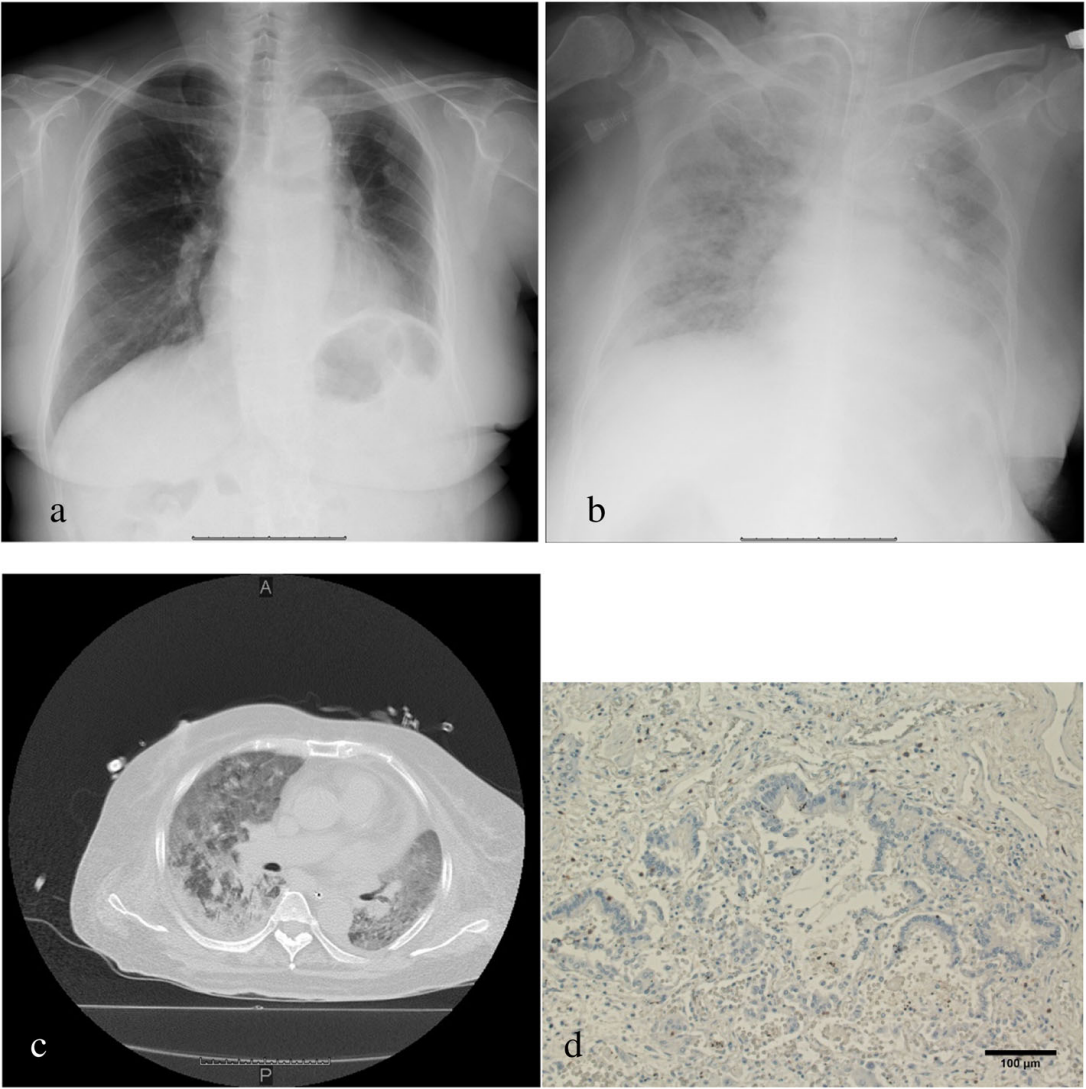
Chest X-ray, computed tomography findings, and immunohistochemistry at autopsy after thrombocytopenia. **a** Chest X-ray on admission for thrombocytopenia shows no noteworthy findings. **b**, **c** Chest X-ray and computed tomography scan at 24 days after admission show reduced bilateral permeability. **d** Immunohistochemistry at autopsy. CD8-positive tumor-infiltrating lymphocytes are focally positive, probably induced by nivolumab

On arrival, she was hemodynamically stable (blood pressure 128/79 mmHg, pulse rate 56 beats/minute, temperature 36.2 °C). There was no abnormal finding on her respiratory sounds. Her cardiac, abdominal, and neurological examinations were also without focal findings. Over time, symptoms such as nasal bleeding and bilateral purpura of her lower limbs developed. Her human leukocyte antigen (HLA) subtype was checked after emergency admission, and it was discovered that she had HLA-DRB1*0405 and DRB0901. Although she underwent a platelet transfusion every day, her platelet count remained low (Fig. 3), and platelet-associated immunoglobulin G (PA-IgG) antibody was relatively high (223 ng/10^7^ cells). She gradually developed hemoptysis and dyspnea because of alveolar hemorrhage. Four days after admission, she required mechanical ventilation, and she developed upper gastrointestinal bleeding, macroscopic hematuria, renal dysfunction, and liver dysfunction. Intravenous immunoglobulin (IVIG) for 7 days, methylprednisolone pulse with maintenance therapy, and romiplostim (recombinant thrombopoietin receptor agonist; TRA) every week were then added to her treatment. Fifteen days after admission, her platelet counts recovered slightly; however, paradoxical cerebral infarction occurred at the left claustrum. Her general condition did not improve despite intensive therapy (Figs. 3, 4); she died 29 days after admission. There was fatal diffuse microscopic bleeding in the lungs, kidneys, pancreas, and ovaries on autopsy. On the other hand, analysis of the lung tumor showed necrotic change, probably induced by nivolumab, because immunohistochemistry showed CD8-positive tumor-infiltrating lymphocytes that were focally positive around the carcinoma (Fig. 4d). Bacterial pneumonia with Gram-positive cocci was also found, but there was no interstitial pneumonia. Based on the above findings, immune thrombocytopenia induced by nivolumab was determined to be the cause of death.

## Discussion and conclusions

A case of severe grade V thrombocytopenia caused by nivolumab in a patient with relapsed NSCLC was reported because this is an educational case and a warning for all physicians and surgeons prescribing ICIs, regardless of the carcinoma. While the pathogenesis of nivolumab-related thrombocytopenia remains uncertain, it is postulated to mimic idiopathic thrombocytopenic purpura (ITP). In the present case, the mechanism of immune thrombocytopenia was likely to have been caused mainly by PA-IgG antibodies produced by activated lymphocytes. The approved treatments for thrombocytopenia most frequently recommended and used are steroids, IVIG, TRAs, platelet transfusion, splenectomy, and other immunosuppressive agents such as azathioprine and rituximab [10]. Only a few cases of nivolumab-induced thrombocytopenia in patients with NSCLC have been reported to date (Table 1) [6–9], although none of these cases was fatal.

**Table 1:** Reported cases of immune-related thrombocytopenia induced by nivolumab in patients with non-small cell lung cancer

Author (reference)	Year	Age (years)/sex	Cycle	PLT lowest count	PA-IgG (ng/10^7^ cells)	Treatment	Other ir-AE	Outcome
Bagley *et al.* [6]	2016	34/M	8	33,000/μL	NR	TRA	None	Recovered
Karakas *et al*. [7]	2017	78/M	6	5000/μL	NR	S	None	Died of cancer
Jotatsu *et al*. [8]	2018	62/M	2	1600/μL	473	S	Hashimoto’s disease	Recovered
Tokumo *et al*. [9]	2018	56/F	3	19,000/μL	NR	S, I	Pancytopenia	Died of cancer
Present case	2018	82/F	2	2000/μL	223	S, I, TRA	None	Died of AE

*AE* adverse event, *F* female, *I* immunoglobulin, *ir-AE* immune-related adverse event, *M* male, *NR* not reported, *PA-IgG* platelet-associated immunoglobulin G, *PLT* platelets, *S* steroid therapy, *TRA* thrombopoietin receptor agonist

The present patient had severe systemic symptoms followed by bleeding from multiple organs and paradoxical cerebral infarction. She did not recover despite intensive therapy including steroid pulse therapy, a TRA, platelet transfusion, IVIG, mechanical ventilation, and so on. Nomura *et al.* reported that patients with ITP who had the HLA-DRB1*0410 allele were extremely resistant to steroid therapy [11]. The present patient did not have the HLA-DRB1*0410 allele, but she had HLA-DRB1*0405, which is the second most frequent allele in patients with ITP who are resistant to steroid therapy. This may explain why she had a weak response to steroid therapy. On the other hand, cerebral infarction in the present case might have been related to the IVIG she received. The pathogenesis of the subsequent brain infarction is thought to involve alteration of blood consistency after many doses of IVIG [12]. For other ICIs, there are also a limited number of reports of immune thrombocytopenia induced by pembrolizumab. Le Roy *et al.* reported two cases of thrombocytopenia in patients with melanoma related to pembrolizumab [13], and there are no reports of thrombocytopenia induced by atezolizumab in the English-language literature. In patients with malignant melanoma, there are also several reports of ICI-induced thrombocytopenia related to nivolumab [14–16].

Pillai *et al*. reported a large-scale systematic comparison of the toxicity profile of PD-1 or PD-L1 inhibitors in patients with NSCLC [17]. In that report, thrombocytopenia was not described as a major AE, and we also recognized ICI-induced thrombocytopenia as a rare AE. Recently, Delanoy *et al*. reported hematological ir-AEs induced by anti-PD-1 or anti-PD-L1 immunotherapy [18]. They reported grade 2 or worse hematological ir-AEs in 35 patients (3.7%), and immune thrombocytopenia was seen in 9 patients (0.9%). Two patients had hemorrhagic symptoms, although grade V thrombocytopenia was not seen. Two patients did not response to steroids or IVIG and were treated with TRAs or rituximab. They concluded that immunological cytopenia is a rare but potentially serious complication of anti-PD-1 or anti-PD-L1 immunotherapies. Another ICI, ipilimumab, which is a cytotoxic T lymphocyte antigen-4 antibody, has been reported to induce immune thrombocytopenia [19]. In most cases treated by ICIs, relatively frequent AEs such as fatigue and diarrhea, or ir-AEs such as hypothyroidism or pneumonitis, could be managed by multidisciplinary treatment, although life-threatening AEs such as immune thrombocytopenia could occur. We have to consider that ICIs are not only long-lasting and effective drugs in patients with NSCLC, but they also have the possibility of causing severe ir-AEs, including thrombocytopenia.

A very difficult case of grade V immune-related thrombocytopenia after the administration of nivolumab as second-line therapy in a patient with relapsed lung adenocarcinoma was described. We should always consider that ICI treatment can lead to AEs that are difficult to predict, and a systematic support framework for ir-AEs and their predictive biomarkers should be established.

## Data Availability

All references may be accessed via hyperlink. No datasets were used in the preparation of this manuscript.

## Abbreviations

AE: Adverse event
ECOG: Eastern Cooperative Oncology Group
EGFR: Epidermal growth factor receptor
HLA: Human leukocyte antigen
ICI: Immune checkpoint inhibitor
ILD: Interstitial lung disease
ir-AE: Immune-related adverse event
ITP: Idiopathic thrombocytopenic purpura
IVIG: Intravenous immunoglobulin
NSCLC: Non-small cell lung cancer
PA-IgG: Platelet-associated IgG
PD-1: Programmed cell death 1
PD-L1: Programmed cell death ligand 1
TRA: Thrombopoietin receptor agonist
